# Microplastic exposure is associated with epigenomic effects in the model organism *Pimephales promelas* (fathead minnow)

**DOI:** 10.1093/jhered/esae027

**Published:** 2024-05-14

**Authors:** Miranda J Wade, Kennedy Bucci, Chelsea M Rochman, Mariah H Meek

**Affiliations:** Department of Integrative Biology, Michigan State University, East Lansing, MI 48824, United States; Ecology, Evolution, and Behavior Program, Michigan State University, East Lansing, MI 48824, United States; Department of Ecology and Evolutionary Biology, University of Toronto-St. George Campus, Toronto, Ontario M5S 3B2, Canada; Department of Ecology and Evolutionary Biology, University of Toronto-St. George Campus, Toronto, Ontario M5S 3B2, Canada; Department of Integrative Biology, Michigan State University, East Lansing, MI 48824, United States; Ecology, Evolution, and Behavior Program, Michigan State University, East Lansing, MI 48824, United States

**Keywords:** DNA methylation, epigenetics, freshwater biodiversity, microplastics

## Abstract

Microplastics have evolutionary and ecological impacts across species, affecting organisms’ development, reproduction, and behavior along with contributing to genotoxicity and stress. As plastic pollution is increasing and ubiquitous, gaining a better understanding of organismal responses to microplastics is necessary. Epigenetic processes such as DNA methylation are heritable forms of molecular regulation influenced by environmental conditions. Therefore, determining such epigenetic responses to microplastics will reveal potential chronic consequences of this environmental pollutant. We performed an experiment across two generations of fathead minnows (*Pimephales promelas*) to elucidate the transgenerational epigenetic effects of microplastic exposure. We exposed the first generation of fish to four different treatments of microplastics: two concentrations of each of pre-consumer polyethylene (PE) and PE collected from Lake Ontario. We then raised the first filial generation with no microplastic exposure. We used enzymatic methylation sequencing on adult liver tissue and homogenized larvae to evaluate DNA methylation differences among treatments, sexes, and generations. Our findings show the origin of the plastic had a larger effect in female minnows whereas the effect of concentration was stronger in the males. We also observed transgenerational effects, highlighting a mechanism in which parents can pass on the effects of microplastic exposure to their offspring. Many of the genes found within differentially methylated regions in our analyses are known to interact with estrogenic chemicals associated with plastic and are related to metabolism. This study highlights the persistent and potentially serious impacts of microplastic pollution on gene regulation in freshwater systems.

## Graphical Abstract

**Graphical Abstract:**
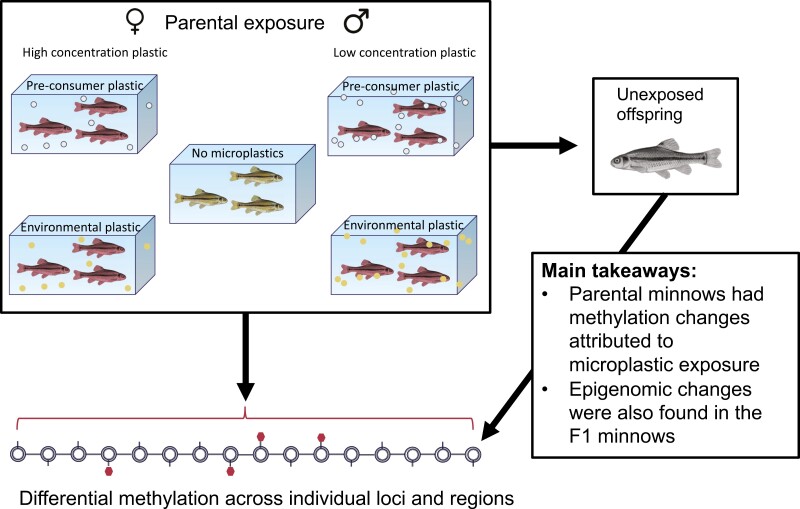

## Introduction

Global microplastic (plastic particles <5 mm in length) pollution is increasingly recognized as a threat to aquatic biodiversity due to its ubiquity, potential impacts across organisms, and persistence (Reid et al. 2018; Corinaldesi et al. 2021). This is a unique challenge of the Anthropocene, as large-scale plastic production began in the early 20th century and has since risen to over 350 million metric tons (Mt) produced annually (Geyer et al. 2017; Napper and Thompson 2020). Much of this plastic enters aquatic environments, estimated at over 20 Mt of waste annually (Borrelle et al. 2020; Chen et al. 2020). To date, records of microplastics exist in marine (Coyle et al. 2020), freshwater (Wagner et al. 2014; Li et al. 2018), atmospheric (Zhang et al. 2020a), and terrestrial (Dissanayake et al. 2022) ecosystems and interact with the biota therein (Anbumani and Kakkar 2018). Given this pervasiveness, it is important we understand the various effects of microplastic exposure on organisms.

Exposure to microplastics is linked to many physiological and ecological effects on aquatic biota. For example, microplastic ingestion negatively affects feeding activity in lugworms (*Arenicola marina*), leading to weight loss (Besseling et al. 2013). Following microplastic exposure, the marine copepod *Tigriopus japonicus* experienced increased oxidative stress (Choi et al. 2020). Multiple instances of uptake, translocation, and genotoxic effects of microplastics are reported in the marine mussel *Mytilus edulis* (Browne et al. 2008; Moos et al. 2012; Avio et al. 2015). In juvenile Chinese mitten crabs (*Eriocheir sinensis*), exposure to polystyrene microplastics resulted in tissue accumulation and reduced growth (Yu et al. 2018). Many studies using zebrafish (*Danio rerio*) found myriad changes related to microplastic exposure, including developmental toxicity and negative reproductive effects (Bhagat et al. 2020). The important commercial fish species European seabass (*Dicentrachus labrax*), Atlantic horse mackerel (*Trachurus trachurus*), and Atlantic chub mackerel (*Scomber colias*) had microplastics accumulated in their gills, dorsal muscle, and gut, as well as higher levels of lipid peroxidation consistent with oxidative damage (Barboza et al. 2020). This evidence across a gamut of species illustrates the many ways in which microplastic particles can affect aquatic organisms.

An additional, concerning feature of microplastics is the ability to sorb persistent organic pollutants (POPs) from the environment (Ziccardi et al. 2016; Sleight et al. 2017; Anbumani and Kakkar 2018; Amelia et al. 2021; Santos et al. 2021), and thereby transmit additional contaminants to organisms (Avio et al. 2015; Paul-Pont et al. 2016; Hal et al. 2020). Prior work suggests the presence of microplastics aggravates the effects of other contaminants when co-exposed (Zhang et al. 2020b). Exposure to microplastics with sorbed chemicals is linked to increased mortality and the reduction of important ecosystem services in lugworms (*A. marina*) (Browne et al. 2013). In marine medaka (*Oryzias melastigma*), exposure to microplastics with sorbed phenanthrene increased bioaccumulation and reproductive toxicity (Li et al. 2022). Zebrafish exposed to microplastics spiked with Benzo[α]pyrene experienced increases in histopathological signs of intestinal inflammation and reduced fecundity (Tarasco et al. 2022). The additional consequences of sorbed chemicals contribute to the complexity of effects in organisms interacting with environmental microplastics (Rochman 2015).

Many studies focus on physiological and ecological effects, but microplastics also affect molecular processes such as gene regulation (Patra et al. 2022). Determining correlations between microplastic exposure and heritable, non-DNA sequence altering, or epigenetic, changes in gene regulation allows us to better understand potential mechanisms behind phenotypic and population-level changes in species (Vandegehuchte and Janssen 2014). As DNA methylation is one such epigenetic response, determining its changes across organisms can have major implications in understanding responses to environmental change or chronic pollutants (Weinhold 2006). Despite this importance, few studies have investigated the epigenetic effects of microplastic exposure. In male rats, polyethylene microplastic exposure increased DNA methylation in a dose-dependent manner (Farag et al. 2023). Exposure to polyethylene microplastics had no significant effect on global DNA methylation levels in *Daphnia magna* (Song et al. 2022), however, individual genes were not analyzed. Chemicals commonly associated with plastics, such as Bisphenol-A, are associated with decreased global methylation in zebrafish (*D. rerio*) (Laing et al. 2016) and phthalates have well-documented epigenetic effects (Dutta et al. 2020). Additionally, epigenetic modification not only affects molecular processes in the exposed animal but can also persist through generations (Vandegehuchte et al. 2010). Studying these responses to microplastic exposure in aquatic environments will provide a better understanding of the potential for long-lasting implications of this pollutant.

Prior research investigated the trans- and intergenerational effects of microplastic exposure in various species. In *D. magna,* parental microplastic exposure was associated with transgenerational effects in growth and reproduction (Song et al. 2022). Similar studies in other aquatic species found parental exposure to microplastics correlated with decreased survival, changes in immunity, delayed development, and reduced locomotor activity in the F1 generation (Yu and Chan 2020; Bringer et al. 2022; Sun et al. 2022). 60-and 150-Day parental exposure scenarios in marine medaka (*O. melastigma*) found changes in hatching, heart rate, and growth/body length in offspring (Wang et al. 2019, 2021). As these studies helped elucidate the presence of important generational impacts due to microplastic exposure, there is a need to investigate the mechanisms behind these alterations. One such method for exploring these mechanisms is through determining epigenetic changes in DNA methylation levels attributed to microplastic exposure, an area ripe for investigation. We addressed these knowledge gaps by studying the epigenetic effects of parental microplastic exposure on offspring using fathead minnows (*Pimephales promelas*), a fish native to the Laurentian Great Lakes region of North America. Our questions included:

1) Are there changes in methylation due to microplastic exposure?
2) How does the effect vary by plastic origin and concentration?
3) Are there transgenerational effects of microplastics on methylation patterns?

To investigate the generational effects of microplastic exposure on methylation patterns, we raised a generation of fish under five treatments: two microplastic concentrations (present and predicted future concentrations) and two types of microplastic [polyethylene (PE) sourced from the manufacturer (“pre-consumer") and PE collected from the beach of Lake Ontario (“environmental”)], and negative control. We raised the offspring of these minnows without microplastic exposure to determine any persistent, transgenerational effects of microplastic exposure on DNA methylation. This approach of evaluating the impacts of microplastic exposure from multiple plastic origins/concentrations and across generations provides insight into the epigenetic effects of microplastics on freshwater organisms. This allows us to better understand the consequences of developing, living, and reproducing in an environment polluted with microplastics.

## Materials and methods

### Experimental design

**Fig. 1.**
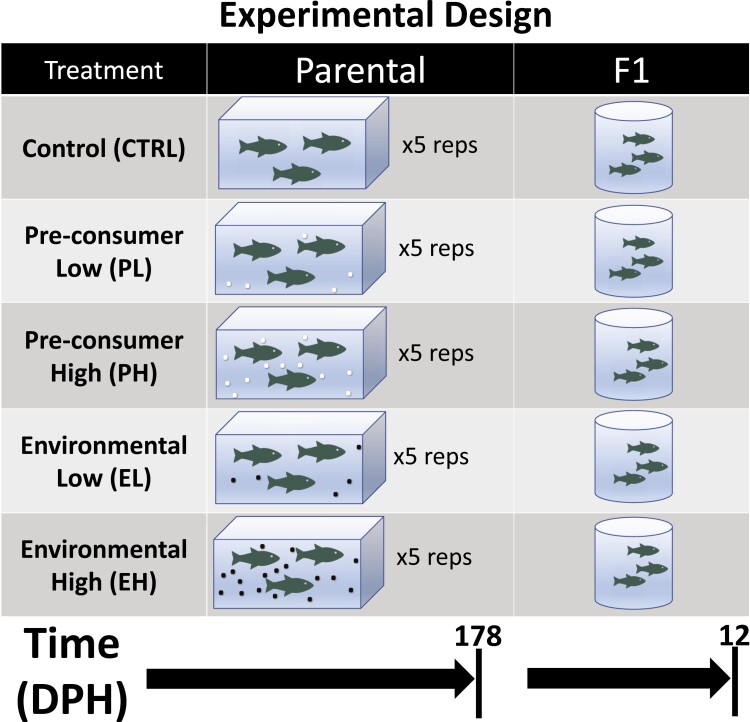
Experimental design. Sampling time points are given below the figure. DPH = days post-hatch. Pre-consumer plastics directly from the manufacturer are depicted with white dots, and environmental plastics from Lake Ontario are depicted with black dots. Each parental treatment had five replicates. Clutches of F1 minnows were collected from each replicate that was reproductively active.

We used fathead minnow, a toxicological model organism, throughout this experiment to determine the effects of microplastics across life stages. Fish maintenance and handling followed protocols approved by the Ontario Ministry of Environment, Conservation, and Parks (MECP) Laboratory Services Animal Care Committee (approval number: ATU-004-19). We adapted rearing methods from a previously published fathead minnow life cycle experiment (Parrott and Blunt 2005) . We obtained fathead minnows from the breeding stock of the MECP. From eggs through 178 days post-hatch (dph), we raised fish under experimental conditions with four treatments and a negative control (CTRL). The treatments were: low, environmentally relevant concentrations (100 particles/L) of 1) pre-consumer PE in the form of black craft pellets (PL) or 2) PE gathered from Lake Ontario (EL) (Grbić et al. 2020); and high, hypothetical future scenario concentrations (2000 particles/L) of 3) pre-consumer PE (PH), or 4) originating in Lake Ontario (EH). We reduced microplastic size to 150 to 500 µm using a burr mill coffee grinder. For a full lifecycle assessment, we used 5 replicate tanks per treatment, for a total of 25 aquaria. We began with 30 fish per tank and destructively sampled to a final count of two male and three female minnows per tank. The experimental design is summarized in Fig. 1. For more details see Bucci et al. 2024.

Each treatment received twice daily feedings. The initial diet consisted of newly hatched brine shrimp at a concentration of 15 nauphii/µL until 30 dph, followed by a gradual introduction of frozen brine shrimp into the diet until 50 dph, after which point the diet consisted solely of thawed brine shrimp. Throughout the experiment, we maintained the water bath at 25 °C and a daily light exposure of 16 h. We completed 30% water changes three times a week and cleaned the aquaria to remove algae buildup and tested a random tank from each treatment for dissolved oxygen, pH, and conductivity to determine water quality. Additionally, tanks contained zeolite to control ammonia levels, and we checked ammonia levels in a sequential manner for each treatment during cleaning.

We encouraged mating by providing egg tiles in each tank. We obtained clutches of offspring from 23 of the 25 tanks as some tanks did not produce eggs or did not lay enough eggs. After the minnows laid eggs on the clay tiles located in each tank, we removed and counted the eggs. If more than 100 eggs were laid, we set 50 aside to raise a second generation of minnows to 12 dph under negative control conditions (no microplastics) to isolate the transgenerational effect of parental exposure. We fed newly hatched brine shrimp to the larvae once daily starting on the second day following the hatching of the first individual. A complete water change was done every other day. The experiment concluded at 178 dph. For final processing, we euthanized fish using a lethal bath of buffered MS-222 at a concentration ≥250 mg/L. We left the fish in the bath until 2 min after respiration ceased. We dissected liver tissue from one male and two females from each tank (*n* = 75) and placed the tissue in 95% ethanol. Between fish, we cleaned dissection tools with 10% bleach, molecular-grade deionized water, and 90% ethanol. For the larvae, entire individuals were placed in ethanol following euthanasia.

### Library preparation and quality filtration

We homogenized the liver tissue of individual adults (*n* = 75) and pooled larvae from familial clutches (*n* = 21) using a bead mill with steel beads prior to extraction. We used a bead-based method to extract and purify genomic DNA (Ali et al. 2016) and the NEBNext Enzymatic Methyl-seq kit (#E7120L) for enzymatic conversion of methylated nucleotides, individual barcoding, and library preparation (New England Biolabs, Inc, Ipswich, MA). This method of methylation detection converts methylated cytosines into a product resistant to deamination, which modifies unmethylated cytosines to uracils and then thymine prior to sequencing. This technique is overall less damaging to DNA and provides single base-pair resolution (Vaisvila et al. 2021). The samples (*n* = 96) were pooled into a single library and sequenced twice on a single lane of SP flowcell (PE 2 × 150) on the Illumina Novaseq6000 at the RTSF Genomics Core at Michigan State University.

Following sequencing reads from both sequencing runs were trimmed using *TrimGalore!* (https://www.bioinformatics.babraham.ac.uk/projects/trim_galore/) to remove adapter-contaminated sequences and the first ten base pairs from the 5ʹ and 3ʹ ends of the fragments. We converted the fathead minnow reference genome (GenBank accession GCA_016745375.1) (Burns et al. 2015; Saari et al. 2017) for methylation alignment using *Bismark* (Krueger and Andrews 2011) and aligned reads to the converted genome using Bowtie2 (Langmead and Salzberg 2012) within *Bismark* with a modified stringency setting of --score_min L,0,−0.6. Alignments were deduplicated from each library using deduplicate_bismark and individuals from each sequencing run merged into a single file using SAMtools version 1.16.1 (Danecek et al. 2021). Prior to further analysis, we removed samples from the dataset with less than 1 million reads. We subsequently extracted methylation calls for individual loci using bismark_methylation_extractor (Krueger and Andrews 2011).

### Relatedness determination

To determine relatedness among individuals, we used the merged and sorted bam files to extract single nucleotide polymorphisms (SNPs) using *BCFtools* mpileup (Danecek et al. 2021) piped to *BCFtools* call (Li 2011). We quality-filtered the resulting file using *VCFtools* v0.1.15 (Danecek et al. 2011), initially removing genotypes not represented in at least 50% of individuals and any SNPs with a count less than five. The resulting data was then further filtered to remove genotypes with fewer than three reads, genotypes with a call rate of less than 90%, and SNPs with a minor allele frequency of less than 0.05. We removed individuals with a missingness greater than 15% and then calculated pairwise φ among all samples using the relatedness2 estimator. Samples with pairwise φ ranges [0.177, 0.354], were denoted as first-degree relatives, which includes full siblings and parent-offspring pairs. Kinship coefficient ranges [0.0884, 0.177] and [0.0442, 0.0884] were considered 2nd-degree and 3rd-degree relationships, respectively (see https://www.kingrelatedness.com/manual.shtml).

### Differential methylation analysis

We used the R packages bsseq (Hansen et al. 2012), dmrseq (Korthauer et al. 2017), DSS (Wu et al. 2013, 2015; Feng et al. 2014; Park and Wu 2016), and MethylSig (Park et al. 2014) to quality filter our methylation data and determine both loci with and regions of differential methylation. Briefly, we read in the coverage files from *Bismark* to a BS-seq object, first filtering out any loci with coverage of less than 10 and then filtering out any individuals with an average read count of less than 0.5. Our final filtration step involved filtering out any loci that did not occur in at least 90% of individuals from the parental generation (*n* = 58) and the F1 generation (*n* = 21). To account for biological variation among our samples, we used the reparametrized model incorporated in DSS (dmlTest, callDML, callDMR) to allow for dispersion in our data (Wu et al. 2015). The stringency values for a locus/region to be called differentially methylated were a delta of 0.1 and a false discovery rate (FDR) of 0.05. We calculated the AM per nucleotide for each individual and then tested for differentially methylated loci (DMLs), defined as a single nucleotide with differential methylation levels between explanatory variables and regions (DMRs), which are regions of the genome with coordinated methylation that are inclusive of multiple DMLs. We tested for differential methylation across the variables of and interactions within sex (female, male), exposure (treated, control), concentration (high, low, control), plastic-type (environmental, pre-consumer, control), individual treatments (environmental high, environmental low, pre-consumer high, pre-consumer low, control), and generation (parental, F1). We used the NCBI Genome Data Viewer for the *P. promelas* annotation release 100 to search for genes containing DMRs (or DMLs if there were no statistically significant DMRs).

## Results

Our libraries averaged >60% genome alignment and >98% cytosine conversion. The average number of merged, aligned, and deduplicated reads per sample was 8,032,241. Following quality filtration, the dataset used to analyze relatedness consisted of 26,463 SNPs across 72 samples. The average missingness among the samples was less than 5%. Unsurprisingly for a laboratory-bred lineage, all minnows analyzed were highly related to one another. Most of the minnows had pairwise relatedness values consistent with first-degree relatives. The average relatedness among all minnows from both generations was 0.368, which is somewhat higher than the range for first-degree relatives. We found no evidence of family effects contributing to gene methylation responses. For the differential methylation analyses, we removed 16 individuals from our final dataset due to poor sequencing (<1 million reads). The final dataset used in differential methylation analysis consisted of 19,338 methylated loci across 79 individuals. Of these, 21 individuals were from the F1 generation and 58 from the parental. When analyzing all samples together, there were no instances of significant differential methylation across the treatments, plastic origin, or plastic concentrations. The only variable with many differentially methylated loci (DMLs) or regions (DMRs) in the combined dataset was between the generations themselves, with 17,615 DMLs and 892 DMRs (see Supplementary Table 1) (Goll and Halpern 2011).

### Parental methylation analysis: female minnows

To account for sex-based differences, we separated the data from the parental minnows by sex. We found a total of 3,651 DMLs and 278 DMRs among the females. The interactions between plastic origin and concentration yielded differential methylation between several treatments. We found 2,046 DMLs and 96 DMRs between the low concentrations of environmental plastic from Lake Ontario and pre-consumer plastics, 538 DMLs and 123 DMRs between the low concentration of environmental plastic and the controls, and 3,020 DMLs and 147 DMRs between the environmental low and pre-consumer high treatments in females (Supplementary Table 1). There were 1,539 DMLs and 73 DMRs between the comparisons of pre-consumer plastic from the manufacturer or environmental microplastic, inclusive of both concentrations. While the high concentration regardless of plastic type did not yield any significant differential methylation in the females, there were 9 DMLs comprising a single DMR between the low concentration of plastic and the controls.

Of the treatments compared to the control, we found 4 DMRs between the pre-consumer plastic regardless of concentration and the controls, 14 DMRs between the EL treatment and the controls, 25 DMRs between the PL and control minnows, and 15 DMRs between the PH and control. A summary of the genes found within these regions as well as the genes found within the DMLs of the minnows exposed to the environmental plastic regardless of concentration can be found in Table 1. A potential gene of interest in the analysis of the EL minnows compared to the controls is the ankyrin repeat domain 31 (*ANKRD31*) gene, which featured decreased methylation in female minnows exposed to low concentrations of environmental plastic [average methylation (AM) = 0.344] compared to the controls (AM = 0.584). Differential expression in *ANKRD31* is associated with chemical and drug-induced liver injury (Davis et al. 2022) and is known to interact with estrogenic-like chemicals associated with plastics (Ali et al. 2014; Lei et al. 2021). Another differentially methylated gene between these comparisons is adenosine deaminase RNA specific B2 (*ADARB2*), which also showed decreased methylation in the minnows exposed to environmental plastic (AM = 0.315 across 4 DMLs), compared to either the pre-consumer (AM = 0.532) or control (AM = 0.52) treatments. *ADARB2* has recorded DNA methylation changes associated with exposure to estrogenic compounds (Jadhav et al. 2017; Awada et al. 2019). We did not find these two genes to be significantly differentially methylated in the male or F1 minnows. Along with these genes, we found 4 instances of DMRs with increased AM within tRNA-coding genes. These DMRs were in the pre-consumer plastic treatment regardless of concentration (*tRNAL-UAG*, AM = 0.503) compared to the control (AM = 0.332), the PL treatment (*tRNAG-GCC*, AM = 0.471) compared to the control (AM = 0.267), and in the PH treatment (*TRNAA-AGC*, AM = 0.522 and another *TRNAG-GCC*, AM = 0.507) compared to the control (AM = 0.337 and 0.317, respectively). TRNAs are key factors in protein synthesis and are thought to be involved in the quality control of RNA (Hori 2014).

**Table 1.** Summary of the changes in methylation levels in genes associated with DMRs in treatment fish compared to the controls.

Generation	Treatment	Gene [with increased (+)/ decreased (−) average methylation]
Female parental (*n* = 34)	Pre	TRNAL-UAG (+)
	EL	LOC120489314 (−), ANKRD31 (−), LOC120473589 (−), LOC120473532 (−), LOC120473590 (−), LOC120473554 (−)
	PL	TRNAG-GCC (+), LOC120473156 (+)
	PH	TRNAA-AGC (+), LOC120467080 (+), TRNAG-GCC (+), LOC120491149 (+)
Male Parental (*n* = 24)	Plastic exposed	LOC120472127 (−)
	Low	TRNAP-AGG (−), TRNAR-UCG (−), LOC120472127 (−), si:ch211-232b12:5 (−), TRNAG-GCC (−), LOC120488227 (−), LOC120491149 (−), LOC120465806 (−), LOC120465808 (−), LOC120465807 (−), LOC120473533 (−), LOC120473665 (−), LOC120484212 (−)
	Pre	LOC120472127 (−), si:ch211-232b12.5 (−), LOC120465808 (−), LOC120465806 (−), LOC120484212 (−)
	PL	TRNAP-AGG (−), LOC120472127 (−), si:ch211-232b12.5 (−), TRNAG-GCC(−), LOC120488227 (−), LOC120465806 (−), LOC120465808 (−), LOC120465807 (−), LOC120473533 (−), LOC120484212 (−)
	EL	TRNAF-GAA (−), LOC120472127 (−), TRNAR-UCG (−), si:ch211-232b12.5 (−), LOC120491149 (−), LOC120465806 (−), LOC120465808 (−), LOC120473533 (−), LOC120473665 (−)
F1 juveniles (*n* = 21)	Pre	TRNAT-UGU (−)
	Low	LOC120469624 (−)
	EL	TRNAF-GAA (+), LOC120475658 (−)
	PL	LOC120481110 (+)

### Parental methylation analysis: male minnows

**Fig. 2.**
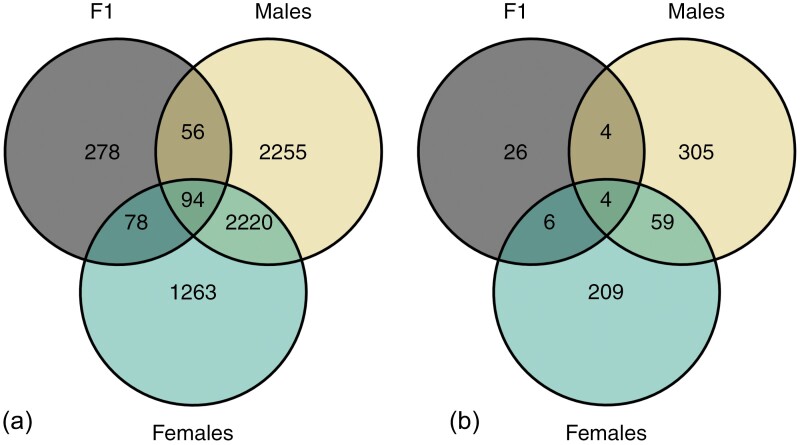
Venn diagrams of the total number of a) DMLs and b) DMRs found among the fathead minnows. The DML Venn diagram is inclusive of those found within DMRs. Male minnows exhibited a greater magnitude of methylation changes than either the females or the F1 generation.

Within the male minnows, we found a total of 4,625 significant DMLs and 372 significant DMRs. Overall, the male minnows exhibited an increased number of DMLs and regions than the females (DMLs = 3,651, DMRs = 278) or F1 minnows (DMLs = 506, DMRs = 40; Fig. 2). Like the females, there was differential methylation attributed to whether the plastic was pre-consumer or environmental, but unlike the females the concentration of the plastic, irrespective of the origin, also elicited a difference in the methylation levels [high compared to low concentration (DMLs = 415, DMRs = 27), control compared to low concentration (DMLs = 395, DMRs = 28); Supplementary Table 1]. There were several comparisons containing large numbers of DMRs and DMLs. These include 2,644 DMRs and 9 DMLs between EH and PL plastic, 1,215 DMLs and 62 DMRs between the EH and pre-consumer plastic regardless of concentration, and 3,786 DMLs and 210 DMRs between the EL and EH plastic from Lake Ontario. We found DMLs and DMRs across genes for transfer RNAs, ribosomal RNAs, uncategorized genes, and regions with no known genes.

Comparisons of methylation levels within various treatments to the controls yielded several genes of potential interest. Overall, there were 13 differentially methylated genes in between the low and control minnows, 5 genes between pre-consumer plastic of any concentration and the controls, 10 genes between the pre-consumer plastic and controls, and 9 genes between the environmental plastic at low concentration and the controls (see Table 1). A gene found across treated minnows regardless of plastic origin or concentration (AM = 0.489) versus controls (AM = 0.719) is Ras-related protein Rab-19 (*LOC120472127*), which is orthologous to *RAB19*, a member of the Ras oncogene family known to interact with estrogenic chemicals associated with plastics (Awada et al. 2019) and a key component of many cellular processes (Jewett et al. 2021). This gene had an average reduction in methylation of 0.23 across the treatments. There was also reduced AM in genes coding for the 5s ribosomal subunit (*LOC120465806*, *LOC120465808*) and translation initiation factor IF-2-like (*LOC120484212*) genes across the low concentration (AM = 0.268, 0.305, and 0.296), pre-consumer plastic (AM = 0.343, 0.348, and 0.295), and PL (AM = 0.251, 0.28, and 0.272), treatments compared to the controls (AM = 0.535, 0.531, and 0.483, respectively). These genes are crucial for ribosomal function.

### Differential methylation analysis: F1 generation

**Fig. 3.**
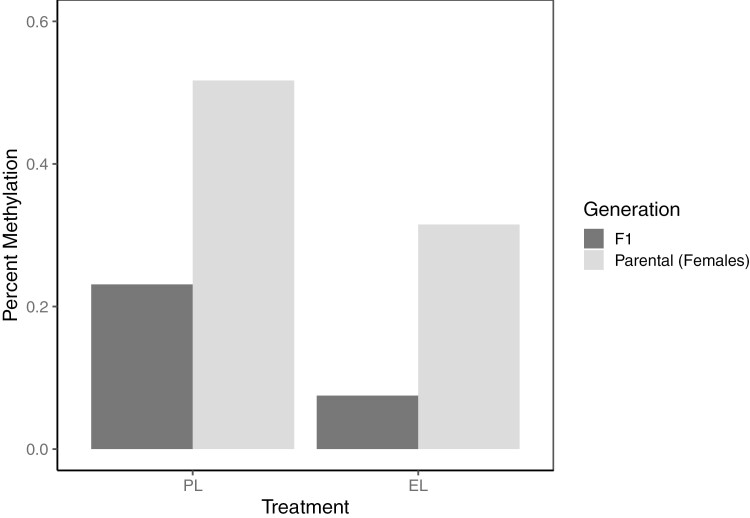
Average methylation levels of a 140-base pair region present on scaffold 7 from nucleotide 19,721,436 to 19,721,575 between the low concentrations of the environmental (EL) and pre-consumer (PL) treatments. Methylation is consistently decreased in the environmental low treatments compared to the pre-consumer low treatments. There were 12 methylated cytosines present in this region, which is found between *TRNAP-AGG* and *TRNAQ-CUG*. Differential methylation was significant between the treatments for each generation. The effects of this methylated region on gene modification or expression are unknown.

Across the F1 juveniles, we found 506 DMLs and 40 DMRs, including a total of 94 DMLs and 4 DMRs shared among the F1, female parental, and male parental minnows (Fig. 2). In DMRs found across both generations of minnows, we found reduced methylation in the environmental plastic compared to the pre-consumer treatments. Three genes within DMRs shared among the F1 and parental minnows were glucocorticoid-induced transcript 1 protein-like (*LOC120481455*), src-like-adapter 2 (*LOC120469624*), and tripeptidyl peptidase 1 (*TPP1*) (Jorgensen et al. 2016; Awada et al. 2019). These genes were differentially methylated between the EL (AM of 0.29, 0.29, and 0.291) and PL (AM = 0.507, 0.518, 0.541) plastic in parental females. This pattern was also found in the F1 minnows, as the offspring of these treatments had reduced AM across these genes (change in AM of −0.19, −0.162, and −0.199; respectively) in the F1 generation. Orthologs of both glucocorticoid-induced transcript 1 protein-like and src-like-adapter 2 have shown methylation changes when exposed to chemicals associated with plastics (Jorgensen et al. 2016; Jadhav et al. 2017). Similarly, *TPP1* has known interactions with estrogenic chemicals (Jorgensen et al. 2016; Awada et al. 2019). We also found shared DMRs in the female and F1 minnows in regions between genes coding for transfer RNAs. An example of this covers a 140 base pair region between tRNAP-AGG and tRNAQ-CUG where there were 12 methylated cytosines (Figure 3). We found overall reduced methylation in this region across both females exposed to low concentrations of environmental (AM = 0.315) compared to pre-consumer (AM = 0.517) plastic and their associated offspring (AM = 0.075 and 0.231, respectively).

**Fig. 4.**
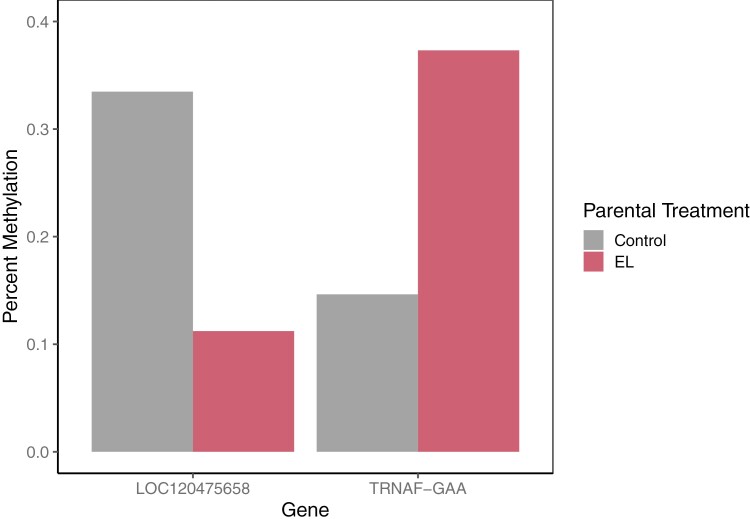
Average methylation percentages of DMRs within the hemicentin-1-like (*LOC120475658*) and transfer RNA (*TRNAF-GAA*) genes in the offspring of parental minnows exposed to either the control (CTRL) or low concentration of environmental origin plastic (EL) treatments. Differential expression in orthologs of *LOC120475658* is associated with necrosis or chemical/drug-induced liver injury. Both DMRs had significant (*P* < 0.05) differential methylation, with changes in average methylation between the control and EL treatments of −0.223 for *LOC120475658* and 0.227 for *TRNAF-GAA*.

Within the F1 analysis, we found 26 DMRs not present in the parental generation. Three of these regions were within genes for the TNF receptor superfamily member 19 (*TNFRSF19*), protein phosphatase 1 regulatory subunit 12A (*PPP1R12A*), and hemicentin-1-like (*LOC120475658*). The region within *TNFRSF19* was significantly less methylated on average between the offspring of minnows exposed to environmental (AM = 0.265) treatments compared to those exposed to pre-consumer treatments (AM = 0.534). *TNFRSF19* is related to development and is known to have differential methylation when exposed to estrogenic chemicals (Jadhav et al. 2017). *PPP1R12A* was differentially methylated between the low concentration of environmental plastic (AM = 0.112) and the high concentration of pre-consumer plastic (AM = 0.327). Previous research has found expression changes in *PPP1R12A* attributed to chemicals associated with plastics in mammals (Ali et al. 2014; Diamante et al. 2021). We found decreased methylation within *LOC120475658* in F1 minnows whose parents were exposed to the low concentration of environmental origin PE (AM of 0.112) compared to those with control parents (AM = 0.335, Fig. 4). Orthologs of *LOC120475658* such as hemicentin-1 (*HMCN1*) are known to respond to estrogenic chemicals related to plastic production (Jadhav et al. 2017; Awada et al. 2019; Olsvik et al. 2019). Finally, similar to findings in the parental generation, we found DMLs/DMRs in 9 tRNA genes (see Fig. 4, Table 1, Supplementary Table 1).

## Discussion

### Are there changes in methylation due to microplastic exposure?

The goal of this study was to increase our understanding of the molecular response to plastic pollution in an important model fish species. Here, we provide evidence for changes in methylation levels across single nucleotides (DMLs) as well as coordinated regions (DMRs) following microplastic exposure. We recorded these effects across the sexes, exposure scenarios, and generations of fathead minnows in our study. Overall, we found more differential methylation in the males of the parental generation compared to both the females and the F1 generation. Many genes within the DMRs of both generations of minnows are related to cellular components and processes (i.e. ribosome-related genes and transfer RNAs), indicating potential differences in cellular metabolism related to exposure to microplastics. Transfer RNAs themselves have a diverse array of cellular functions outside of their key role in translation, (Berg and Brandl 2021), including regulating the cellular response to amino acid starvation (Hinnebusch 2005). Additionally, there is evidence of methylation-induced tRNA dysregulation in complex diseases such as cancer (Rosselló-Tortella et al. 2022; Pinzaru and Tavazoie 2023), so the tRNA methylation changes found throughout this study may relate to a negative response to microplastic exposure. It is unclear, however, what kind of gene-regulatory effect the differential methylation of ribosome-related genes such as 5s ribosomal RNA has since DNA methylation may not affect transcription by certain RNA polymerases, such as RNA polymerase III, in these genes (Besser et al. 1990). Therefore, the biological significance of our observed differential methylation levels among these genes is unknown.

In the second generation of minnows, the effects across microplastics treatment were largely in regions distinct from those in the parental minnows, which is likely due to the different tissue types (adult livers, whole larvae) sampled and developmental stage of the minnows (Goll and Halpern 2011; Klughammer et al. 2023). There were relatively even numbers of DMLs/DMRs found in treatments of different plastic origins and concentrations, however many of these were in areas of the genome with no known genes. However, as the fathead minnow genome continues to undergo updates and improvements, these loci and regions may be eventually described. The presence of F1 DMLs/DMRs associated with the parental treatments, provides evidence toward a multigenerational additive effect, at least in younger life stages. Our study confirming changes in gene methylation provides additional molecular context that complements evidence for changes in enzymatic activity, RNA expression, and histopathology correlated with microplastic exposure (Patra et al. 2022).

### How does the effect vary by plastic origin and concentration?

If the gene methylation levels in the minnows responded differently between the pre-consumer and environmental treatments, this could provide evidence for plastic with sorbed contaminants affecting organisms differently than microplastics without additional contaminants. While the literature on the transference of these chemicals and their subsequent effects is often contradictory (Gassel and Rochman 2019; Rochman et al. 2019), previous work shows PE sorbs greater concentrations of certain organic contaminants than other plastic types (Rochman et al. 2013). This includes work linking the sorption of environmental contaminants to greater hepatic stress in Japanese medaka (*Oryzias latipes*) that ingested PE microplastic (Rochman et al. 2013). Alternatively, the pre-consumer PE likely contains chemical additives as they were craft pellets and dyed black (Hahladakis et al. 2018), which may cause different effects in organisms compared to environmental plastics. An example would be the potential presence of plasticizers like phthalates, which have known endocrine-disrupting properties in aquatic species (Mathieu-Denoncourt et al. 2015). Similarly, a larger response to higher concentrations of microplastics may imply more egregious effects in organisms as microplastic levels rise. Alternatively, exposure to lower concentrations may cause greater methylation change, similar to the low-dose effect commonly associated with endocrine-disrupting chemicals (Vandenberg et al. 2012).

Interestingly, we observed subtle differences in gene methylation levels attributed to changes in plastic concentration or plastic origin (pre-consumer or environmental). The nature of these changes differed between the male and female individuals. Female minnows experienced the largest magnitude of DMRs and loci in comparisons related to plastic origin. This finding is complementary to work in developing zebrafish (Tarasco et al. 2022), larval fathead minnows (Bucci et al. 2022), and adult Japanese medaka (*O. latipes*) (Rochman et al. 2013) where environmental or chemical-spiked microplastics elicit a greater response than pre-consumer plastics. As many of the genes within these DMLs/regions in the females are known to interact with estrogenic chemicals that leach from plastics (Yang et al. 2011; Bittner et al. 2014), the differences in the concentrations of these chemicals between pre-consumer microplastics and those exposed to the environment likely attribute to this observed difference in relative methylation.

Contrastingly, we found a greater number of DMLs and DMRs across the plastic concentrations in the male minnows, indicating males may have a greater sensitivity to microplastics concentration than females, or perhaps that females have a similar response to microplastics regardless of concentration. Some of these differences may be linked to changes in the allocation of resources to reproduction when stressed. As fathead minnows exhibit a “fast” or r-selected life history characterized by prioritizing reproduction over survival (Stearns 1977), the differences in response may be in part because fathead minnows exhibit polygyny and thus the males have a higher stress response than females (Tarka et al. 2018). This result coincides with previous work suggesting dose-dependent effects of microplastics on rainbow trout (*Oncorhynchus mykiss*) (Roch et al. 2022), dose-dependent reduction in male fecundity following microplastic exposure in Japanese medaka (Zhu et al. 2020), and research suggesting male Chinese mitten crabs are increasingly sensitive to microplastic exposure as concentration increases (Sun et al. 2022). However, the latter work was in the testis specifically, whereas our findings are liver-specific, so future work investigating the methylation changes in gonads would further characterize the differences in sensitivity between males and females. As other studies have shown that male fish exhibit a greater inflammatory response (Procópio et al. 2014) or increased hepatic damage (Guillante et al. 2023) related to pollutant exposure than females, there are likely many sex-dependent changes in organisms when exposed to microplastics. Overall, our findings provide more evidence that microplastics act as complicated stressors with multiple effects, including the response to the physical presence of microplastics themselves and the additive effect of the sorbed chemicals.

### Are there transgenerational effects of microplastics on methylation patterns?

As we raised the F1 generation under the same conditions as the control, seeing similar methylation patterns in the F1 minnows across the parental treatment types may implicate chronic effects of microplastics that can persist through generations. Inter- and transgenerational inheritance of methylation patterns may impact gene transcription and thus disrupt many molecular processes (Vandegehuchte et al. 2010). For example, if multigenerational methylation changes are in genes involved in basic cellular metabolism, as we have shown here, this may lead to epigenetically inherited compensation or tolerance to microplastics with yet-unrecorded physiological and population consequences (Vandegehuchte and Janssen 2014). For the most part, we observed a lesser magnitude of differential methylation in the offspring compared to the parental generation (Table 1, Supplementary Table 1). Overall lesser methylation in juveniles is not an unexpected result given previously recorded variations in methylation across development that often feature lesser methylation overall (Goll and Halpern 2011).

Interestingly, the generations shared few genes within DMRs/DMLs. As we compared whole larvae from the F1 generation to liver samples from the parental generation, there are likely more methylation changes found throughout other tissue types in the adult minnows, especially in the gonads, that we were unable to capture with this experimental design. Genes with shared differential methylation throughout the F1 juveniles included transfer RNAs, transmembrane proteins, and genes related to inflammation and necrosis pathways, many of which are known to interact with plastic-related chemicals. We found a greater magnitude of differential methylation in the F1 juveniles from the interactions of plastic origins and concentration than from either variable independently, which supports the presence of an increased effect due to the complicated interaction of concentration and plastic origin (Rochman et al. 2019; Bucci et al. 2020, 2022; Mei et al. 2020). Our gene-level methylation findings expand upon findings of inter- and transgenerational reproductive toxicity but not global methylation changes in *D. magna* following microplastic exposure (Song et al. 2022) and serve as some of the first evidence of DNA methylation changes attributed to microplastics, as most epigenetic work to date has featured nanoplastics (López de las Hazas et al. 2022). Given that fish species such as salmonids develop methylation changes to shifts in their environment associated with domestication (Barreto et al. 2019; Koch et al. 2023), and exposure to toxic hydrogen sulfide results in inherited methylation changes in the shortfin molly *Poecilia mexicana* (Kelley et al. 2021), our findings provide evidence that similar epigenetic changes attributed to plastic pollution may occur in fish.

### Implications

Our study presents evidence that exposing even a single generation of fish to microplastics can have chronic and multigenerational methylation effects, even once the microplastic exposure is removed. As altered methylation patterns can indicate rapid, intergenerational yet reversible reactions to environmental changes (Vandegehuchte and Janssen 2011, 2014), our findings help anchor the molecular response to microplastics to the potential ecological and evolutionary implications of DNA methylation. Additionally, this is some of the first epigenetics work outside of nanoplastics and in a fish species native to the Laurentian Great Lakes. In an ecotoxicological context, there remains a knowledge gap in the transmittance of pollutant-induced epigenetic changes and the subsequent ability to induce microevolutionary changes (Medina et al. 2007). Since previous work reveals a correlation between patterns of DNA methylation and genome-wide divergence (Herrera and Bazaga 2016), these kinds of analyses are necessary in understanding the effects of persistent, chronic pollutants such as microplastics and predicting future implications. Studying mechanisms of differential gene expression along physiological timescales (such as exposure scenarios) helps shape hypotheses along evolutionary timescales such as predicting population changes in response to chronic pollution (Chevin et al. 2010; Reid and Whitehead 2016; Shama et al. 2016; Garant 2020). As the rapid, repeated evolution of tolerance to toxins has been reported in Killifish (Reid et al. 2016), further investigating links between DNA methylation changes and plastic pollution is a key direction for future work. This study provides information on the persistent impacts of microplastic pollution that could impact freshwater environments even beyond future restoration efforts, increasing our knowledge of the molecular responses to anthropogenic pollution. Predicting future effects of microplastics pollution using a model organism is an ideal method of identifying the negative, and potentially enduring, effects of plastic pollution and informing plans to mediate the effects of plastic pollution.

## Supplementary Material

Supplementary material is available at *Journal of Heredity* Journal online.

esae027_suppl_Supplementary_Table

## Data Availability

We have deposited the primary data underlying these analyses as follows: Sample metadata and initial genomic data object (.Robj) to Dryad.
