# Supplementary material for: Microplastic exposure is associated with epigenomic effects in the model organism Pimephales promelas (fathead minnow)
Source: J Hered. 2024 May 14;116(2):113–25. doi: 10.1093/jhered/esae027 (PMC11879203; doi:10.1093/jhered/esae027)
Supplement: esae027_suppl_Supplementary_Table [file esae027_suppl_supplementary_table.docx]

| Supplementary Table 1. Summary of differentially methylated loci (DMLs) and differentially methylated regions (DMRs) from DSS analysis of differential methylation with a false discovery rate (FDR) <0.05 and a minimum delta of 0.1. Abbreviations are environmental plastic (Env), pre-consumer plastic (Pre), control (Ctrl), low concentration (Low), high concentration (High), environmental low (EL), environmental high (EH), pre-consumer low (PL), pre-consumer high (PH). | | | | |
| --- | --- | --- | --- | --- |
| **Dataset** | **Comparison** | **DMLs** | **DMRs** | **Genes (if known)** |
| All (n=79) | Parental, F1 | 17615 | 892 | Not investigated |
| Parental (n=55) | Male, Female | 65 | 3 | LOC120473533, tRNA (n=1) |
|  | Env, Pre | 4 | -- | -- |
|  | High, Low | 11 | 1 | tRNA (n=1) |
|  | EL, Ctrl | 4 | -- | -- |
|  | EL, PH | 158 | 11 | tRNA (n=3), LOC120467080, LOC120491149, LOC120465807 |
|  | PH, PL | 4 | -- | -- |
|  | EL, PL | 43 | 1 | -- |
|  | EL, EH | 50 | 3 | -- |
| Female parental (n=34) | Env, Pre | 1539 | 73 | LOC120489314, tRNA (n=12) ANKRD31, LOC120475989, GRIA4B, LOC120491149, LOC102465807, NPFFL, LOC120473156, LOC120473586, LOC120473585, LOC120473590, LOC120473532, LOC120473533, LOC120473555, LOC120475309, LOC120475315, ADARB2, zmp:0000000936, LOC120481455, LOC120486186 |
|  | Env, Ctrl | 9 | -- | ADARB2, LOC120475832 |
|  | Pre, Ctrl | 84 | 4 | tRNA (n=1) |
|  | Low, Ctrl | 9 | 1 | -- |
|  | EL, Ctrl | 538 | 13 | LOC120489314, ANKRD31, LOC120473589, LOC120473532, LOC120473590, LOC120473554 |
|  | PL, Ctrl | 10 | 2 | tRNA (n=1), LOC120473156 |
|  | PH, Ctrl | 173 | 15 | tRNA (n=2), LOC120467080, LOC120491149 |
|  | EH, PL | 147 | 13 | LOC120475989, tRNA (n=1), LOC120473156 |
|  | EH, PH | 473 | 32 | LOC120472127, tRNA (n=3), LOC120491149, LOC120465807, NPFFL, GPM6BB, zmp:0000000936, LOC120486186 |
|  | EL, PH | 3020 | 147 | LOC120486679, LOC120489314, ARHGAP17B, APOL, tRNA (n=23), LOC120467080, ARHGEF12B, ANKRD31, PKD1A, TBX4, LOC120480528, SI:CH211-232B12.5, *acsl2*, *cavin4b*, LOC120491149, INO80DB, ZGC:165604, LOC120460519, LOC120461512, MMP14A, LOC120465807, *NPFFL*, LOC120473156, LOC120473588, LOC120473533, LOC120473584, LOC120473586, LOC120473589, LOC120473554, LOC120473585, LOC120473590, LOC120473555, LOC120473533, LOC120475315, LOC120475309, LOC120475320, GJB7, ADARB2, GPM6BB, LOC120484212, LOC120484214, LOC120486186 |
|  | EL, PL | 2046 | 96 | LOC120486679, TPP1, LOC120489314, ARHGAP17B, tRNA (n=21), LOC120467080, ARHGEF12B, ANKRD31, LOC120469624, LOC120475989, ZGC:165604, LOC120461512, LOC120473156, LOC120473586, LOC120473532, LOC120473554, LOC120473533, LOC120473588, LOC120473585, LOC120473590, LOC120473555, ADARB2, LOC120481455, LOC120486186 |
|  | EL, EH | 231 | 13 | tRNA (n=1), ANKRD31, LOC120473554 |
| Male parental (n=24) | Ctrl, Plastic exposed | 56 | 6 | LOC120472127 |
|  | Env, Pre | 11 | 1 | -- |
|  | Env, Ctrl | 1 | -- | -- |
|  | Pre, Ctrl | 126 | 11 | LOC120472127, si:ch211-232b12.5, LOC120465808, LOC120465806, LOC120484212 |
|  | High, Low | 415 | 27 | tRNA (n=6), LOC120491149, LOC120465807, LOC120465806, LOC120465808, LOC120473156, LOC120473555 |
|  | Low, Ctrl | 395 | 28 | tRNA (n=3), LOC120472127, si:ch211-232b12:5, LOC120488227, LOC120491149, LOC120465806, LOC120465808, LOC120465807, LOC120473533, LOC120473665, LOC120484212 |
|  | EL, Ctrl | 397 | 22 | tRNA (n=2), LOC120472127, si:ch211-232b12.5, LOC120491149, LOC120465806, LOC120465808, LOC120473533, LOC120473665 |
|  | EH, Ctrl | 1 | -- | -- |
|  | PL, Ctrl | 354 | 25 | tRNA (n=2), LOC120472127. si:ch211-232b12.5, LOC120488227, LOC120465806, LOC120465808, LOC120465807, LOC120473533, LOC120484212 |
|  | PH, Ctrl | 14 | 1 | -- |
|  | PH, PL | 67 | 3 | tRNA (n=1), LOC120465807, LOC120465806, |
|  | EH, PL | 2644 | 139 | LOC120489314, LOC120489314, TSPAN2A, LOC120463284, tRNA (n=26), SFTPBB, ARHGEF12B, PKD1A, LOC120459336, LOC120471030, LOC120475989, LOC120481110, LOC120488227, LOC120491149, LOC120491149, INO80DB, SHANK2A, LOC120459923, LOC120461512, MMP14A, LOC120465807, LOC120465806, LOC120465808, LOC120468055, LOC120473156, LOC120473589, LOC120473532, LOC120473555, LOC120473585, LOC120473590, LOC120473533, LOC120473554, LOC120475309, LOC120475315, GPM6BB, LOC120484212, LOC120484213, LOC120484212, LOC120484214 |
|  | EH, PH | 1215 | 62 | LOC120489314, LOC120489968, LOC120463284, tRNA (n=10), SOUL5L, PKD1A, LOC120459336, LOC120475989, LOC120481110, LOC120486626, SI:CH211-57I17.5, MMP14A, SI:DKEY-172J4.3, NPFFL, LOC120473264, LOC120473589, LOC120473532, LOC120473588, LOC120473585, LOC120473590, LOC120473555, LOC120473533, LOC120475309, LOC120475315, GPM6BB, LOC120484212, LOC120484214, |
|  | EL, PH | 14 | -- | -- |
|  | EL, PL | 2 | -- | -- |
|  | EL, EH | 3786 | 210 | LOC120489314, LC120489968, tspan2a, LOC120463284, tRNA (n=17), sftpbb, LOC120459336, LOC120471010, LOC120471019, tbx4, acsl2, LOC120481110, LOC120486626, LOC120488227, LOC120491149, SI:CH211-57I17.5, MMP14A, TMEM145, LOC120465807, LOC120465806, LOC120465808, LOC120468055, LOC120473264, LOC120473586, LOC120473533, LOC120473588, LOC120473532, LOC120473589, LOC120473554, LOC120473532, LOC120473555, LOC120473585, LOC120473665, LOC120475315, LOC120475309, GJB7, GPM6BB, ZMP:0000000936, LOC120484212, LOC120484213, LOC120484212, LOC120484214, LOC120485500, |
| F1 juveniles (n=21) | Env, Pre | 12 | -- | TNFRSF19 |
|  | Env, Ctrl | 17 | -- | TNFRSF19 |
|  | Pre, Ctrl | 33 | 3 | tRNA (n=1) |
|  | High, Low | 24 | 2 | -- |
|  | High, Ctrl | 14 | 1 | -- |
|  | Low, Ctrl | 59 | 4 | LOC120469624 |
|  | EL, Ctrl | 109 | 9 | tRNA (n=1), LOC120475658 |
|  | EH, Ctrl | 39 | 2 | -- |
|  | PL, Ctrl | 97 | 6 | LOC120481110 |
|  | PH, Ctrl | 24 | 2 | -- |
|  | PH, PL | 96 | 6 | TPP1, LOC120481110, NPFFL |
|  | EH, PL | 93 | 8 | TPP1, TNFRSF19, LOC120468725, LOC120481455 |
|  | EH, PH | 24 | 1 | -- |
|  | EL, PH | 88 | 6 | PPP1R12A |
|  | EL, PL | 119 | 9 | TPP1, tRNA (n=1), LOC120481110 |
|  | EL, EH | 119 | 9 | tRNA (n=5), LOC120481110, LOC120475658 |
